# Quantitative and qualitative analysis of bioaerosols emissions from the domestic eastern wastewater treatment plant, Alexandria, Egypt

**DOI:** 10.1038/s41598-024-79645-z

**Published:** 2024-12-16

**Authors:** Ebtesam El-Bestawy, Mohammed Mahmoud Ibrahim, El sayed Ahmed Shalaby

**Affiliations:** Department of Environmental Studies, Institute of Graduate Studies & Research (IGSR), Alexandria University, 163 Horria Ave. El-Shatby, P.O. Box 832, Alexandria, Egypt

**Keywords:** Air sampling, Bioaerosols, Wastewater treatment plants, 16S rDNA sequencing, Biological techniques, Microbiology, Climate sciences, Environmental sciences, Risk factors

## Abstract

Bioaerosol studies showed that wastewater treatment plants (WWTPs) are a significant source of bioaerosol emissions. In this study, 170 samples of total bacteria, total coliform, and total fungi were collected from 10 sites within a domestic WWTP, Alexandria, Egypt, using the sedimentation technique. According to the Index of Microbial Air Contamination (IMA) classes, the total bacteria range was 108–5120 CFU/dm^2^/hour, and all samples were classified as “very poor” except one sample of an office, which was classified as “poor.” The total coliform range was 0–565 CFU/dm^2^/hour, and 6 samples were classified as “very poor,” while one sample was classified as “poor.” The total fungi range was 0–209 CFU/dm^2^/hour, and 9 samples were classified as “very poor,” while 4 samples were classified as “poor.” After the conversion to CFU/m^3^, the counts of total bacteria, total coliforms, and total fungi were 897 − 42.7 × 10^3^, 0–4.71 × 10^3^, and 0–2.69 × 10^3^ CFU/m^3^, respectively. Several identified bioaerosols have been reported before as a cause of human infections. They included *Lysinibacillus fusiformis*,* Bacillus cereus*,* Alcaligenes faecalis*,* Klebsiella* sp., *Escherichia coli*,* Aspergillus* spp., *Penicillium* spp., *Rhizopus* sp., *Candida* sp., and *Rhodotorula* sp. These results indicated an increased health risk to WWTP staff, which needs more attention and more efficient control measures.

## Introduction

Air pollution alone causes about 7 million deaths a year, ranking it among the top global risks to health^1^. Air pollutants can be classified as non-biological particles, biological particles, gases, and physical pollutants^2,3^. Any airborne particles derived from biological sources, such as bacteria, viruses, fungus, plants, animals, and protozoa, are referred to as bioaerosols^4^. Even though bioaerosols represent a small fraction of all aerosol particles in our surroundings, their effects can be crucial as a single viable microbial pathogen could be sufficient to spread infection^5^. It’s a multidisciplinary study area that comprises fields such as microbiology, mechanical engineering, air pollution, medical science, epidemiology, immunological science, biochemistry, nanotechnologies, etc^6^. A significant knowledge gap still exists till now in the scientific understanding of the physical characteristics and monitoring of bioaerosols, and more studies are needed covering developing countries^7,8^.

Human exposure to bioaerosols has been correlated with a range of acute and chronic adverse health effects and diseases that are related to toxic and allergenic materials, and to infectious diseases from pathogenic biological agents. Respiratory infections (e.g. rhinitis, asthma, bronchitis and sinusitis), which often can be linked to inhaled pathogens, are the most severe diseases worldwide in terms of mortality. Other health problems reported include digestive disorders, fatigue, weakness and headache^5,9^. Recently, COVID-19 (as a type of bioaerosols) caused 7,043,660 deaths worldwide till 31 March 2024^10^.

The number of methods available for the collection of microorganisms is roughly equal to the number of investigators^11^. These numerous methods included conventional methods and real-time samplers. On the other hand, the absence of an accurate, rapid, easy, and inexpensive method for quantifying bioaerosols remains a barrier in the bioaerosols assessment. Therfore, choosing the appropriate method for bioaerosols monitoring remains a challenge^8^. Sedimentation is one of the commonly used methods in which microorganisms settle directly on the agar medium under the influence of gravity^12,13^. Cartwright et al.^14^ stated that sedimentation cannot used for quantitative monitoring as the volume of sampled air is not determined. Likewise, Michalkiewicz^15^ and Viani et al.^16^ reported that this technique give an overestimation of the risk. Contrariwise, its reliability was examined by comparing fungi counts collected by sedimentation, impingement, and filtration methods^17^. Furthermore, Manibusan & Mainelis^18^ suggested that using of this technique alongside active collection devices can assist researchers in developing countries for comprehensive understanding of biological presence and dynamics. Several studies have applied sedimentation technique for quantitative bioaerosols monitoring in different environments^13,19–23^. In addition, Korzeniewska et al.^24^, Gotkowska-Plachta et al.^25^, and Aborawash et al.^26^ used this technique for sampling and quantifying bioaerosols from wastewater treatment plants (WWTPs). For identification of bioaerosols; despite the fact that morphological characters for bacteria identification are few and limiting^27^, identification based on morphology is a primary step for classifying fungal pathogens at the genus level^28^. In few decades, identifying bacterial isolates in the clinical laboratory by proteomics and sequence rather than phenotype has dramatically improved the diagnostic and epidemiological capabilities of clinical microbiology laboratories^29^.

Extensive review of bioaerosols studies revealed that WWTPs represent a significant source of bioaerosols emissions and workers of WWTPs are exposed to these bioaerosols through various pathways such as ingestion, skin or mucosal contact, inhalation, and contact with contaminated surfaces, clothes or tools etc. WWTPs associated bioaerosols have emerged as one of the critical sustainability indicators, ensuring health and well-being of societies and cities^30^. Therefore, the main objective of this study was to quantify and identify total bacteria, and total fungi in the air of a domestic WWTP and describe their characteristics. Also, total coliform was measured as it reflects the level of air pollution with bioaerosols from sewage around WWTPs^15^.

## Materials and methods

### Sampling sites

**Fig. 1:**
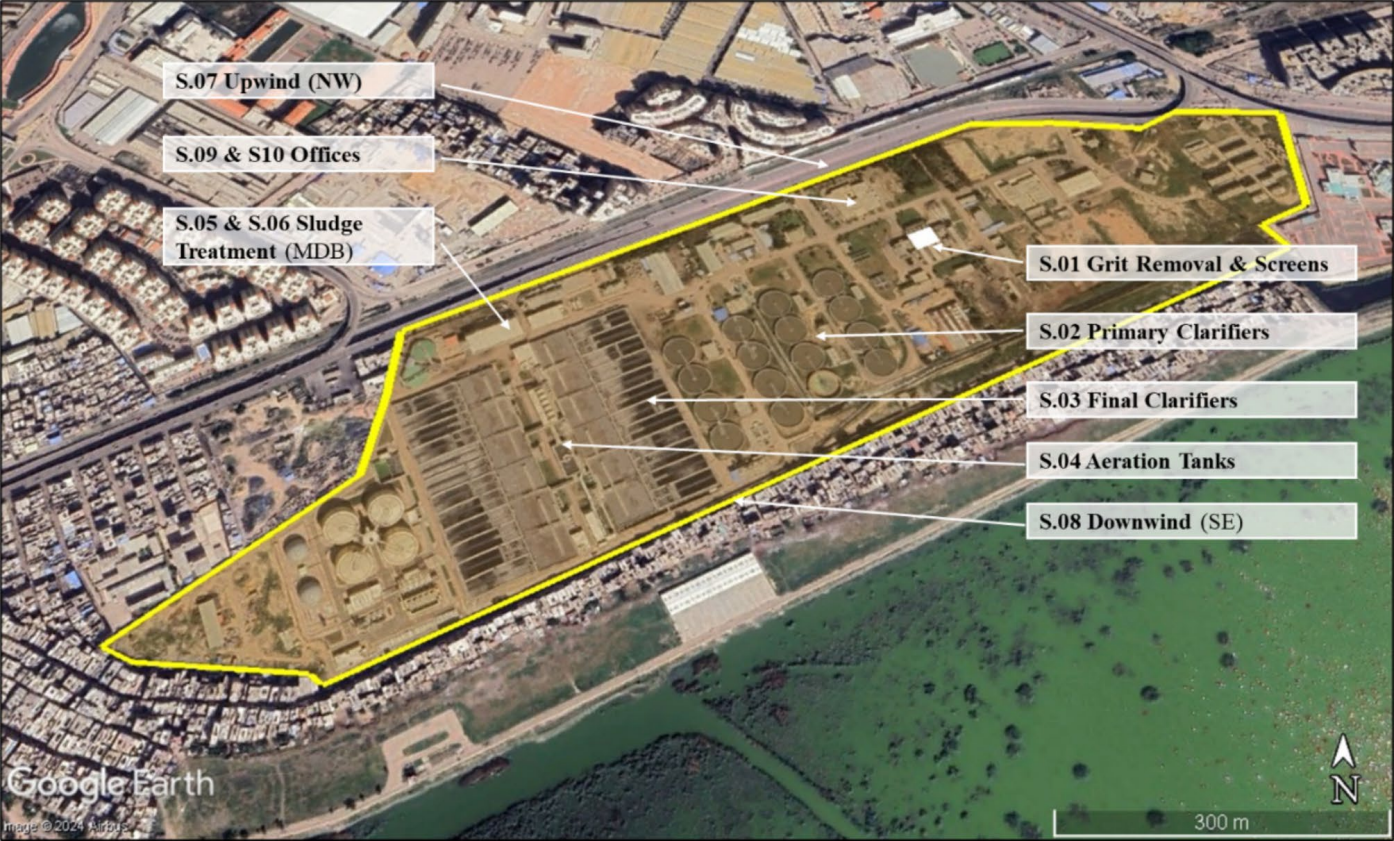
Bioaerosols sampling sites in the eastern WWTP, Alexandria, Egypt (MDB: Mechanical Dewatering Building, NW: Northwest, SE: Southeast).

Alexandria Governorate has 20 WWTPs^31^, and this study aims to assess the risks of bioaerosols emitted from the biggest WWTP in Alexandria (Eastern Wastewater Treatment Plant). This WWTP has a capacity of 800,000 m^3^/day (1,040,000 m^3^/day as peak flow). The process consists of pretreatment and primary treatment (5 screens followed by 10 grit and grease removal chambers then 16 primary clarifiers), 12 modules for biological treatment (1 aeration tank + 2 clarifiers for each module), sludge treatment (thickening and dewatering), and chlorination unit^32^. Total bacteria (total culturable mesophilic bacteria), total coliform, and total fungi (total culturable fungi) were monitored in 10 sites within the WWTP (Fig. 1); grit removal and screens (outdoor, 1st floor between screens and grit removal chambers), primary clarifiers (outdoor, on the edge of the tank), final clarifiers (outdoor, on the upper way between tanks), aeration tanks (outdoor, on the upper way between tanks), sludge treatment (indoor, 1st floor and ground floor), upwind (outdoor), downwind (outdoor), and 2 offices (indoor).

### Samples preparation

Samples were collected on 90 mm Petri dishes containing different types of media, nutrient agar (NA) (HIMedia, India) for total bacteria (28 g was dissolved in 1 L of distilled water and then sterilized by autoclaving at 15 lbs pressure “121 °C” for 15 min), M-Endo agar (HIMedia, India) for total cform (25.5 g was dissolved in 490 ml of distilled water and heated to boiling without autoclaving, then 10 ml of ethanol 95% are added), and sabouraud 4% dextrose agar (SDA) (Merck, Germany) for total fungi (32.5 g was dissolved in 500 g of distilled water, 50 mg of Chloramphenicol (NEOGEN, USA) was added to inhibit bacterial growth, then sterilized by autoclaving at 15 lbs pressure “121 °C” for 15 min).

### Field sampling

Total bacteria, total coliform, and total fungi were sampled 5–6 times in 10 sites (170 samples as a total) during November and December 2021 using the sedimentation technique during day working hours (mainly between 9:00 am and 02:00 pm). The average sampling time was 10 min, and the samples were taken at the height of 100: 145 cm (close to/within the breathing zone for standing workers) in 8 sites, while samples in offices were taken at the height of 75 cm (within the breathing zone for seated workers). Samples were transported between the sampling site and the lab in an icebox, and the transportation time didn not exceed 40 min for any trip. Meteorological data was available online from Alexandria station (HEAX) which is located about 2 km from the WWTP. During the 6 sampling campaigns, temperature range was (16.7–27.15 °C), relative humidity range was (53.67–77.25%), wind speed range was (1.6–3.6 m/s), and precipitation was (0 mm).

### Laboratory analysis

**Fig. 2:**
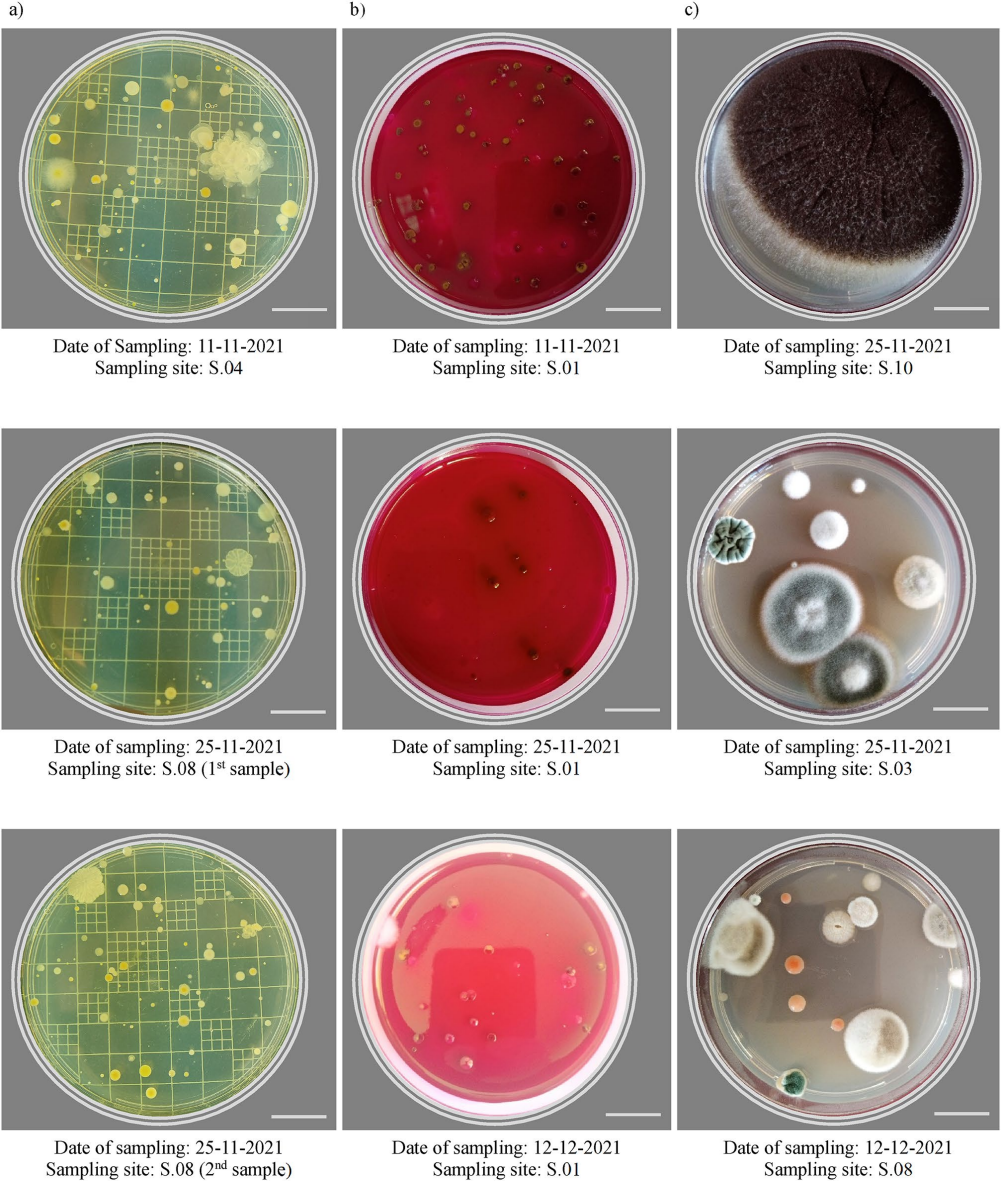
Different samples after incubation (a) total bacteria colonies on nutrient agar (48 h), (b) total coliform colonies on m-Endo agar (48 h), and (c) total fungi colonies on SDA (5 days). Scale bar = 1.8 cm.

Total bacteria and total coliform were incubated for about 48 h (at 36 ± 1 °C) and grown colonies (Fig. 2-a and b) were enumerated using a manual colony counter after 24 and 48 h, while total fungi were incubated for 5 days (at 28 ± 1 °C) and colonies were enumerated every day as some colonies may cover the whole plate after the 5 days (Fig. 2-c). Results were expressed as CFU/dm^2^/hour and then compared to the index of microbial air contamination classes (IMA class) as 0–9 CFU/dm^2^/h (very good), 10–39 CFU/dm^2^/h (good), 40–84 CFU/dm^2^/h (fair), 85–124 CFU/dm^2^/h (poor), and ≥ 125 CFU/dm^2^/h (very poor)^33,34^.

In addition, results were expressed as CFU/m^3^ using omeliansky’s formula: *N* = 5 a × 10^4^ (bt)^−1^ where, N is the count in CFU/m^3^, a is th number of colonies per Petri dish, b is the Petri dish area in cm^2^, t is the exposure time in minutes^35^. There is no uniform international standard available up till now for acceptable counts of bioaerosol^36,37^. Table 1 shows suggested limits that were used to compare the study results.

**Table 1:** Suggested thresholds for bioaerosols.

Bioaerosols	Acceptable limits	References
Total bacteria	4500 − 10,000 CFU/m^3^	^13,38^
	750 CFU/m^3^ (museums–indoor)	Italian Ministry of Cultural Heritage (MiBAC) thresholds ^37,39^
	500 CFU/m^3^ (indoor environment)	American Conference of Governmental Industrial Hygienist thresholds (ACGIH) ^40^, WHO thresholds ^36^, and Swedish thresholds ^41,42^
Total coliform	Not Available	
Total fungi	1000 CFU/m^3^ (non-industrial indoor locations)	European Union ^36,43^
	300 CFU/m^3^ (indoor environment)	Swedish thresholds ^41,42^
	150 CFU/m^3^ (museums–indoor)	Italian Ministry of Cultural Heritage (MiBAC) thresholds ^39^

Isolated bacterial colonies were identified using 16 S rRNA gene sequencing. Firstly, the total DNA of isolated bacterial colonies was extracted from samples using Quick-DNA™ Fungal/Bacterial Microprep Kit (Zymo Research, USA). 50–100 mg (wet weight) bacterial cells have been re-suspended in up to 200 µl of water and added to a ZR BashingBead™ Lysis Tube (0.1 mm & 0.5 mm). 750 µl BashingBead™ Buffer was added to the tube. The tube was secured in a bead beater fitted with a 2 ml tube holder assembly and processed at maximum speed for ≥ 5 min then it was centrifuged in a microcentrifuge at 10,000 x g for 1 min. 400 µl of the supernatant was transferred to a Zymo-Spin™ III-F Filter in a Collection Tube and centrifuged at 8000 x g for 1 min. 1200 µl of Genomic Lysis Buffer was added to the filtrate in the Collection Tube then 800 µl of the mixture transferred to a Zymo-Spin™ IICR Column3 in a Collection Tube and centrifuged at 10,000 × g for 1 min. The flow through from the Collection Tube was discarded and the previous step was repeated. Add 200 µl DNA Pre-Wash Buffer was added to the Zymo-Spin™ IICR Column in a new Collection Tube and centrifuged at 10,000 × g for 1 min. 500 µl g-DNA Wash Buffer was added to the Zymo-Spin™ IICR Column and centrifuged at 10,000 × g for 1 min. Finally, the Zymo-Spin™ IICR Column was transferred to a clean 1.5 ml microcentrifuge tube and 100 µl DNA Elution Buffer was added directly to the column matrix and centrifuged at 10,000 × g for 30 s to elute the DNA. Then, The PCR amplification reaction was performed using COSMO PCR RED Master Mix (willowfort, UK). 25 µl of COSMO PCR RED Master Mix and 1–2.5 µl of Forward and Reverse Primers (20µM) were mixed in a nuclease free Eppendorf. Nuclease-free water was added to 50 µl. The master mix was aliquoted into separate 0.2mL PCR tubes before adding the DNA template. The components were mixed gently and centrifuged briefly. The PCR reaction program was set under the following PCR conditions: 95 °C for 2 min “for stage 1”, Primer Tm < 5 °C for 20 s, 72 °C for 30–60 s “for stage 2 and repeated 25–35 cycles”, then 72 °C for 1 min “stage 3”.

Finally, the forward and reverse sequences were refined and merged using BioEdit, version 7.2.5^44^, and the identity of bacterial isolates was assigned by comparing their DNA sequences with those available in the GenBank NCBI (National Center for Biotechnology Information) database using a BLAST+^45^, version: 2.13.0 using the following settings: Models (XM/XP) and (Uncultured/environmental sample sequences) were excluded, Megablast (Results were optimized for highly similar sequences), and results with unspecified species were excluded. Phylogenetic and molecular evolutionary analyses were conducted using MEGA version 11^46^. Coliform and fungi colonies were identified only to the genus level according to the macroscopic morphological characteristics.

## Results and discussion

### Enumeration of bioaerosols

According to IMA classes, total bacteria ranged from 108 to 5,120 CFU/dm^2^/hour and all samples were classified as “very poor” (≥ 125 CFU/dm^2^/hour) (except an office on the 1st day of sampling which was classified as “poor”). Total coliform ranged from 0 to 565 CFU/dm^2^/hour and 6 samples were classified as “very poor” while one sample was classified as “poor”. Total fungi ranged from 0 to 209 CFU/dm^2^/hour and 9 samples were classified as “very poor” while 4 samples were classified as “poor”.

**Fig. 3:**
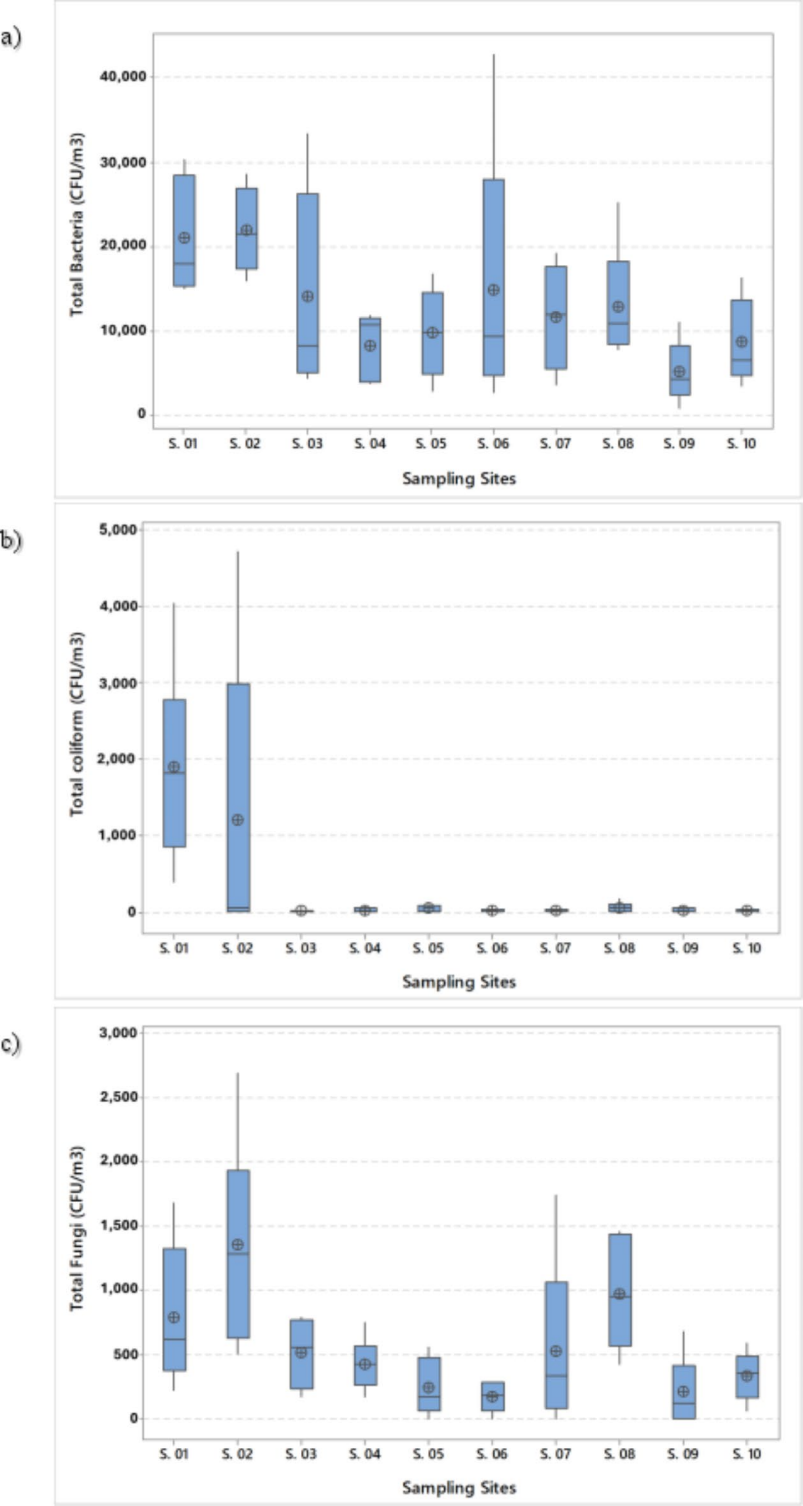
Box plot for (a) total bacteria counts, (b) total coliform counts, and (c) total fungi counts per each sampling site (vertical boxes representing approximately 50% of the observations, whiskers extending from the box roughly represent the upper and lower 25% of the distribution, median values indicated by the horizontal line inside the box, and a circle-cross symbols represent the means).

After the conversion of CFU/plate to CFU/m^3^ using omeliansky’s formula, the count of total bacteria, total coliform, and total fungi varied as shown in Table 2; Fig. 3a-c. Twenty eight samples recorded counts of total bacteria higher than all suggested thresholds (> 10^4^ CFU/m^3^) and Grit removal & screens phase and primary clarifiers recorded the highest mean, while one of the administration offices recorded the lowest mean. For total coliform, the primary clarifiers and grit removal phase & screens recorded the highest mean, while other sites recorded limited contamination with coliform. For total fungi, 8 of 59 samples recorded levels higher than all suggested thresholds (> 1,000 CFU/m^3^), and samples of primary clarifiers recorded the highest mean, while the ground floor of the mechanical dewatering building (sludge treatment) recorded the lowest mean.

**Table 2:** Count of total bacteria, total coliform, and total fungi in different sites of the studies WWTP.

Sampling sites	Total bacteria (CFU/m^3^)				Total coliform (CFU/m^3^)				Total fungi (CFU/m^3^)			
	Min	Max	Mean	St. Dev.	Min	Max	Mean	St. Dev.	Min	Max	Mean	St. Dev.
S. 01	15.0 × 10^3^	30.3 × 10^3^	21.2 × 10^3^	6.89 × 10^3^	392	4.04 × 10^3^	**1.9 × 10** ^**3**^	1.28 × 10^3^	224	1.68 × 10^3^	794	545
S. 02	16.0 × 10^3^	TNTC	**21.9 × 10** ^**3**^	5.18 × 10^3^	ND	4.71 × 10^3^	1.2 × 10^3^	1.96 × 10^3^	505	2.69 × 10^3^	**1.35 × 10** ^**3**^	810
S. 03	4.3 × 10^3^	33.3 × 10^3^	14.1 × 10^3^	12.18 × 10^3^	ND	84	14	34	168	785	512	263
S. 04	3.7 × 10^3^	11.8 × 10^3^	8.3 × 10^3^	4.0 × 10^3^	ND	58	19	29	168	748	428	203
S. 05	2.8 × 10^3^	16.7 × 10^3^	9.7 × 10^3^	5.29 × 10^3^	ND	252	49	101	ND	561	238	219
S. 06	2.7 × 10^3^	42.7 × 10^3^	14.9 × 10^3^	15.99 × 10^3^	ND	168	28	69	ND	280	*168*	113
S. 07	3.6 × 10^3^	19.1 × 10^3^	11.6 × 10^3^	6.4 × 10^3^	ND	84	*17*	38	ND	1.74 × 10^3^	527	694
S. 08	7.7 × 10^3^	25.2 × 10^3^	12.8 × 10^3^	7.07 × 10^3^	ND	168	61	63	421	1.46 × 10^3^	971	454
S. 09	897	11.0 × 10^3^	*5.1 × 10* ^*3*^	3.69 × 10^3^	ND	84	23	37	ND	673	208	258
S. 10	3.5 × 10^3^	16.3 × 10^3^	8.6 × 10^3^	5.07 × 10^3^	ND	168	28	69	56	589	336	188

TNTC: too numerous to count (excluded in calculating the mean and the standard deviation), ND: Not Detected.

The highest count in bold. The lowest count in italics.

**Fig. 4:**
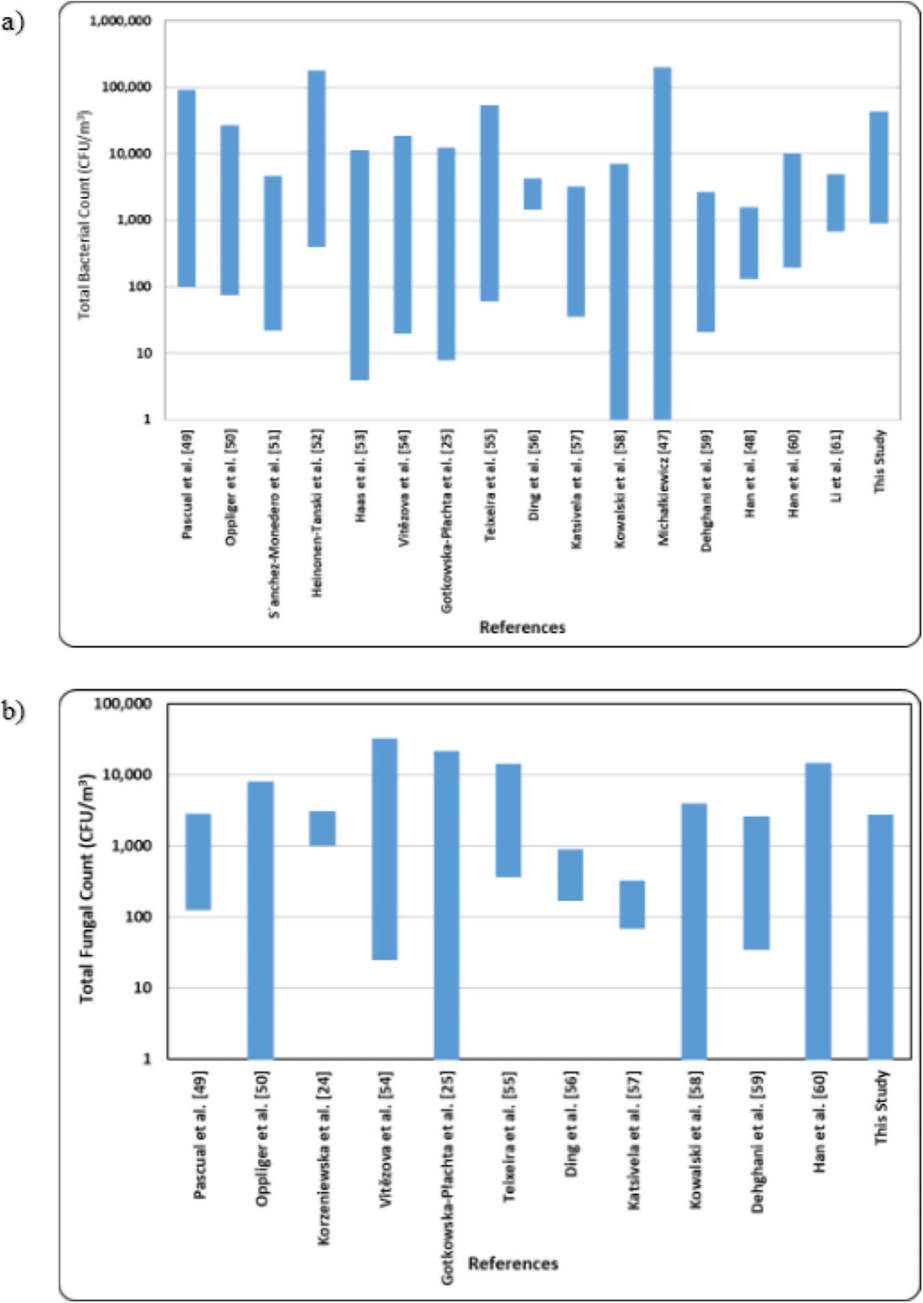
Ranges of (a) total bacteria and (b) total fungi reported in this study compared to previous studies.

For comparison, the findings of this work revealed that total bacteria count ranged between 897 and 4.27 × 10^4^ CFU/m^3^. Total bacteria count ranges varied widely in the previous studies (Fig. 4-a). Michałkiewicz^47^ described that total bacteria count from 11 Municipal WWTPs in Poland was within range from 0 to 1.95 × 10^5^ CFU/m^3^. Conversely, Han et al.^48^ displayed total bacteria count generated in the air from sludge dewatering houses of 9 WWTP in China ranged only between 132 ± 7 and 1.53 × 10^3^ ± 155 CFU/m^3^.

This wide variation might be because of heterogeneity among sampling methods and WWTPs features such as:

- *Number of sampling points within WWTPs*: Heinonen-Tanski et al.^52^, Han et al.^48^, and Haas et al.^53^ reported their results only from one sampling site within the WWTPs (Total bacteria ranges: 400 –1.76 × 10^5^, 132 ± 7–1.53  × 10^3^ ± 155, and 4–1.1 × 10^4^ CFU/m^3^, respectively. On the contrary; Dehghani et al.^59^ sampled total bacteria and total fungi in 25 sites within the WWTP and reported a range from 21 ± 3 to 2.58 × 10^3^ ± 401. In this study, 10 sites were selected to represent all stages.
- *Sampling instruments*: In this study, the sedimentation method was used for bioaerosols sampling, while Gotkowska-Plachta et al.^25^ used sedimentation and impaction methods and recorded a range of 1.2–8.0 × 10^4^ CFU/m^3^. Heinonen-Tanski et al.^52^ used a bio-sampler liquid impinger and recorded 0.4 × 10^3^ − 1.76 × 10^5^ CFU/m^3^. Furthermore, Han et al.^48^ collected samples on a quartz microfiber filter via a suction pump and reported a range from 132 ± 7 to 1.53 × 10^3^ ± 155 CFU/m^3^. Others depended on Six-stage Andersen impactors or Single-stage impactors and reported various ranges.
- *Sampling duration*: Sánchez-Monedero et al.^51^ reported their results for 3 replicates per each sampling site (22–4.58 × 10^3^ CFU/m^3^). Kowalski et al.^58^ conducted a sampling campaign for only one day (0–6.9 × 10^3^ CFU/m^3^). In this study, sampling campaigns were conducted during November and December. On the other hand, Gotkowska-Plachta et al.^25^ collected samples for two annual cycles in spring, summer, autumn, and winter (1.2–8.0 × 10^4^ CFU/m^3^).
- *Incubation conditions*: A big variation in incubation temperatures and time was noticed. In this study, bacteria were incubated at ≈ 37 °C for 48 h as followed in several studies. Contrariwise, Ding et al.^56^ and Han et al.^48^ incubated bacteria at 30 °C for 48 h (1.48 × 10^3^ ± 434 − 4.16 × 10^3^ ± 550, 132  ± 71–1.53 × 10^3^ ± 155 CFU/m^3^, respectively), Oppliger et al.^50^ incubated bacteria also at 30 °C but for 7 days (2.62–75.0 × 10^4^ CFU/m^3^). Furthermore, Gotkowska-Plachta et al.^25^ incubated bacteria at 26 °C for 72 h (1.2–8.0 × 10^4^ CFU/m^3^). In addition, total bacteria were incubated at 20 °C for 3–4 days^53^, and for 7–8 days^49^, and various ranges were reported (ND − 6.9 × 10^3^, 0.4 × 10^3^–1.76 × 10^5^ CFU/m^3^, respectively).
- *Distance from the source*: Kowalski et al.^58^ captured total bacteria close to the wastewater effluent (ND – 6.9 × 10^3^ CFU/m^3^). As well as in this study. Gotkowska-Plachta et al.^25^ took all samples downwind (approximately 1 to 1.5 m from the source) and reported a range from 1.2 to 8.0 × 10^4^ CFU/m^3^. Furthermore, Sánchez-Monedero et al.^51^ conducted outdoor sampling 2–10 m downwind of the operations (22–4.58 × 10^3^ CFU/m^3^).
- *Capacity of WWTPs*: In this study, the WWTP normal capacity is 800,000 m^3^/day (1,040,000 m^3^/day as peak flow)^32^, while Michałkiewicz^47^ sampled bioaerosols from 11 WWTPs with capacities ranging from 350 to 200,000 m^3^/day and reported a range of 0–1.95 × 10^5^ CFU/m^3^. On the contrary, Ding et al.^56^ performed their study in a WWTP with a capacity of 100 m^3^/day only and the range was from 1.48 × 10^3^ ± 434 to 4.16 × 10^3^ ± 550 CFU/m^3^.
- *Design of WWTPs*: WWTPs varied widely in design; (1) Operations may be outdoor or indoor, uncovered or shaded or partially shaded, (2) Indoor phases may be equipped with ventilation system or not, (3) sedimentation tanks and aeration tanks may be constructed above or under the ground level, etc. In this study, the grit removal phase was partially shaded, while primary clarifiers, aeration tanks, and final clarifiers were constructed outdoors without shading.

For the total coliform, the findings of this study revealed that the count ranged from 0 to 4.7 × 10^3^ CFU/m^3^. These results were consistent with the study of Pascual et al.^49^ who reported that the count of total coliform in a municipal WWTP in Spain was within a range of 0 and 3.1 × 10^2^ CFU/m^3^. In the same study^49^, the pretreatment phase recorded the highest count of total coliform (35–3190 CFU/m^3^ with a median of 205 CFU/m^3^) followed by primary clarifiers (20–1165 CFU/m^3^ with a median of 149 CFU/m^3^). In addition, Haas et al.^53^ described that total coliform count generated from three WWTPs in Austria was within a range of 0 and 4.4 × 10^2^ CFU/m^3^. Furthermore, Katsivela et al.^57^ reported a very low count of total coliform in the grit removal phase only (29 ± 27 CFU/m^3^) and Michałkiewicz^47^ displayed that total coliform count generated from 11 municipal WWTP in Poland ranged between 0 and 8.8 × 10^4^ CFU/m^3^.

For the total fungi, the findings of this work revealed that the count ranged between 0 and 2.69 × 10^3^ CFU/m^3^. Total fungi count ranges varied widely in the previous studies (Fig. 4-b). Vítězová et al.^54^ described that total fungi count generated from WWTPs in Czech were within a range of 25 and 3.2 × 10^4^ CFU/m^3^. Conversely, Katsivela et al.^57^ displayed total fungi count generated in a WWTP in Greece ranged only between 70 and 204 CFU/m^3^. This variation might be attributed to the same variables mentioned above.

## Identification of bioaerosols

### Identification of isolated bacteria

**Fig. 5:**
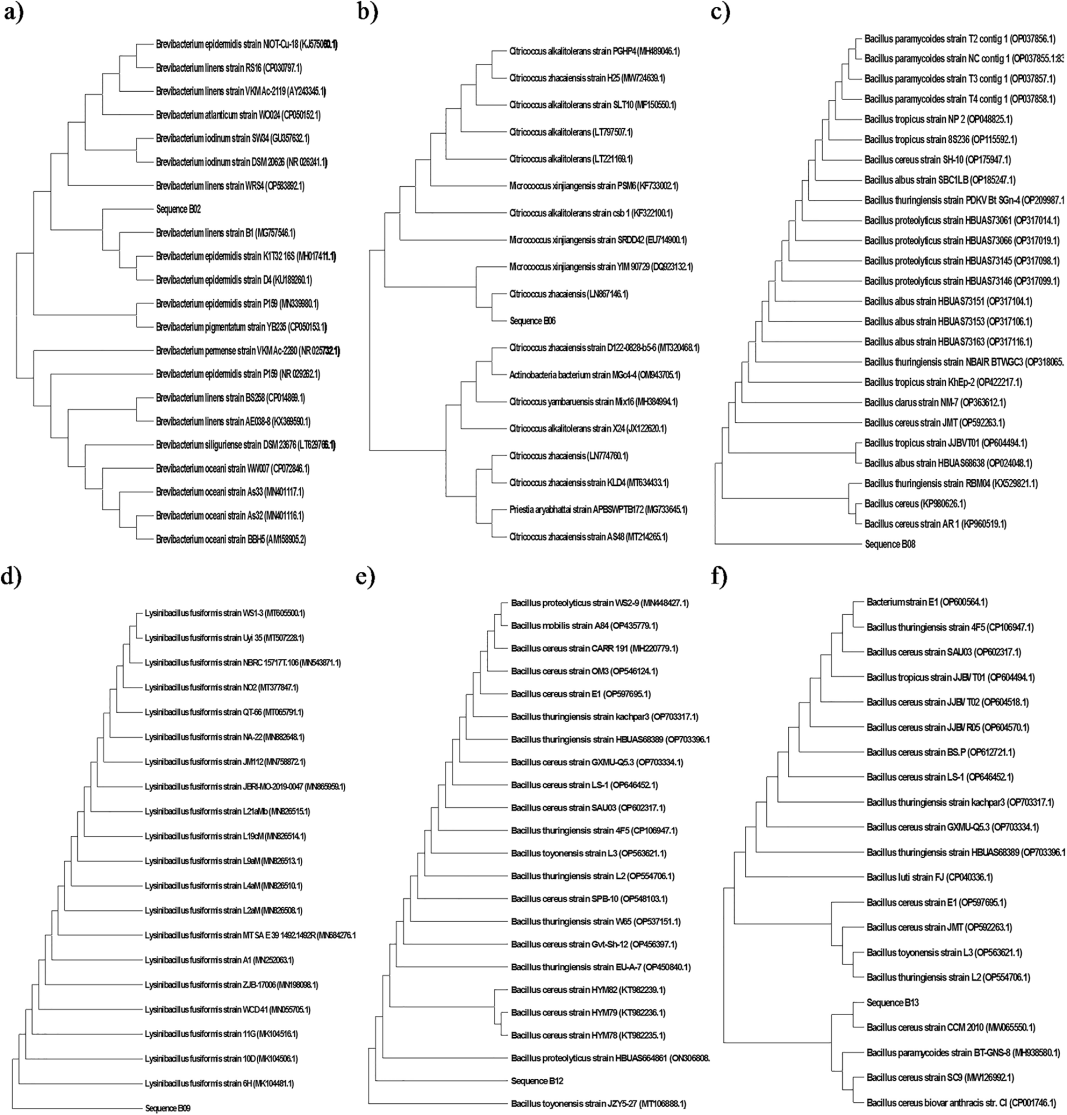

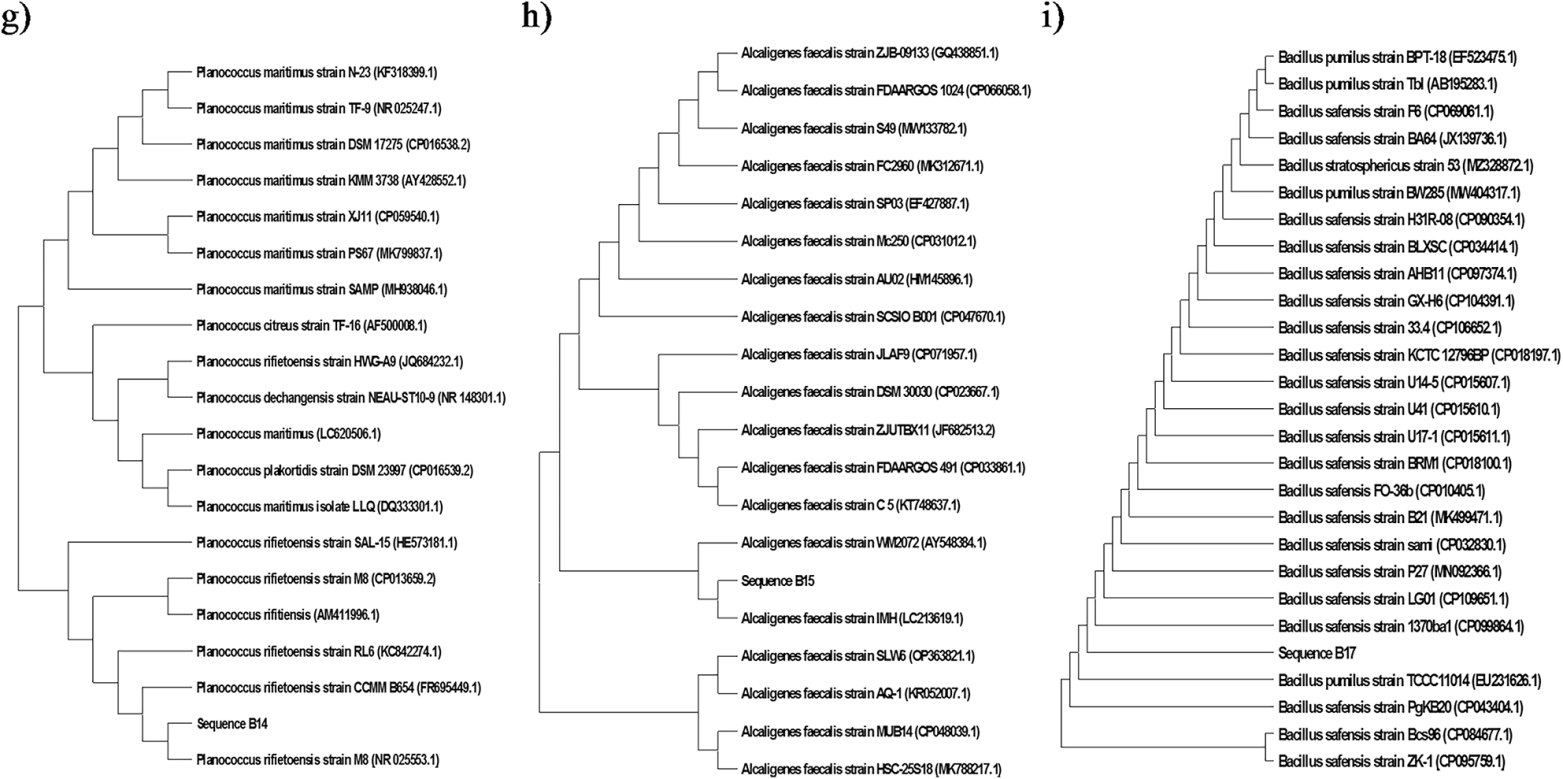
Phylogenetic trees of the dominant bacterial isolates at the eastern WWTP (Alexandria, Egypt), (a) *Brevibacterium linens* strain B01 (PQ164272), (b) *Citricoccus zhacaiensis* strain B06 (PQ164273), (c) *Bacillus* sp. strain B08 (PQ164282), (d) *Lysinibacillus fusiformis* strain B09 (PQ164283), (e) *Bacillus* sp. strain B12 (PQ164289), (f) *Bacillus cereus* strain B13 (PQ164339), (g) *Planococcus rifietoensis* strain B14 (PQ164341), (h) *Alcaligenes faecalis* strain B15 (164346), and (i) *Bacillus safensis* strain B17 (PQ164347).

Twenty bacterial colonies were isolated from collected samples within the domestic WWTP. The 16 S rDNA sequences of the dominant 9 bacterial colonies (B2, B6, B8, B9, B12, B13, B14, B15, and B17) were extracted partially and compared with those available in the GenBank NCBI database using a BLAST+. These isolates identified as *Brevibacterium linens* (B02), *Citricoccus zhacaiensis* (B06), *Bacillus* sp. (B08), *Lysinibacillus fusiformis* (B09), *Bacillus* sp. (B12), *Bacillus cereus* (B13), *Planococcus rifietoensis* (B14), *Alcaligenes faecalis* (B15), and *Bacillus safensis* (B17) as shown in Fig. 5.

The phylogenetic tree of *Brevibacterium linens* (B02) showed a strong similarity with *B. epidermidis* which was isolated formerly from nitrocellulose-contaminated wastewater environments^62^. The phylogenetic tree of B06 (*Citricoccus zhacaiensis*) showed a strong similarity with *Micrococcus xinjiangensis*. *Micrococcus* sp. was also isolated previously from WWTPs^60,63^. The phylogenetic trees of strains B08 and B12 showed similarity with several species of the *Bacillus* genus. Several *Bacillus* spp. were isolated previously from the air of WWTPs^63,64^. B09 (*L. fusiformis*) is recognized as a ubiquitous environmental bacterium and has been isolated from wastewater, plants, and soil. Also, *L. fusiformis* is believed to cause tropical ulcers^65^. *Bacillus cereus* (B13) was isolated previously from the air of a WWTP^66^. *B. cereus* is well known as a cause of food poisoning and much more is now known about the toxins produced by various strains of this species^67^. B14 was identified as *Planococcus rifietoensis* which was reported as a carotenoid pigment producer and as a bio-remediator of paper pulp mill effluent, in an economically and industrially important way^68^. *Alcaligenes faecalis* (B15) usually causes opportunistic infections in humans. Its infection is often difficult to treat due to its increased resistance to several antibiotics. The most frequent *A. faecalis* infection sites, in order, are the bloodstream, urinary tract, skin and soft tissue, and middle ear^69^.

These results confirmed that there is a high degree of a complete similarity across the length of 16 S rRNA for many microorganism groups that don’t allow identification to the species level for some or all species within certain genera^29^. This limitation of 16 S rRNA sequencing was reported early by Fox et al.^70^ by comparing 16 S rRNA sequences for *Bacillus globisporus* W25^T^ (T = type strain) and *Bacillus psychrophilus* W16A^T^, and W5. These strains exhibited more than 99.5% sequence identity and within experimental uncertainty could be regarded as identical. Consequently, 16 S rRNA gene identity may not be sufficient to guarantee species identity.

### Identification of isolated coliform

According to morphological characteristics of coliform colonies stated in the technical data of M-endo agar (HiMedia Laboratories, India), *Escherichia coli* was abundant in all samples (pink colonies with metallic sheen), *Klebsiella* sp. appeared in a few numbers in some samples (pink to red colonies), and no *Salmonella sp.* colonies were observed (colorless to very light pink colonies). Both *E. coli* and *Klebsiella* sp. were isolated previously from the air of WWTP^24,71–73^. *E. coli* is the most common pathogen leading to uncomplicated cystitis, and causes other extra-intestinal illnesses, including pneumonia, bacteremia, and abdominal infections such as spontaneous bacterial peritonitis^74^. Besides, *Klebsiella* represent a severe pathogen that cause pneumonia, bacteremia, thrombophlebitis, urinary tract infection, cholecystitis, diarrhea, upper respiratory tract infection, wound infection, osteomyelitis, and meningitis^75^.

### Identification of isolated fungi

**Fig. 6:**
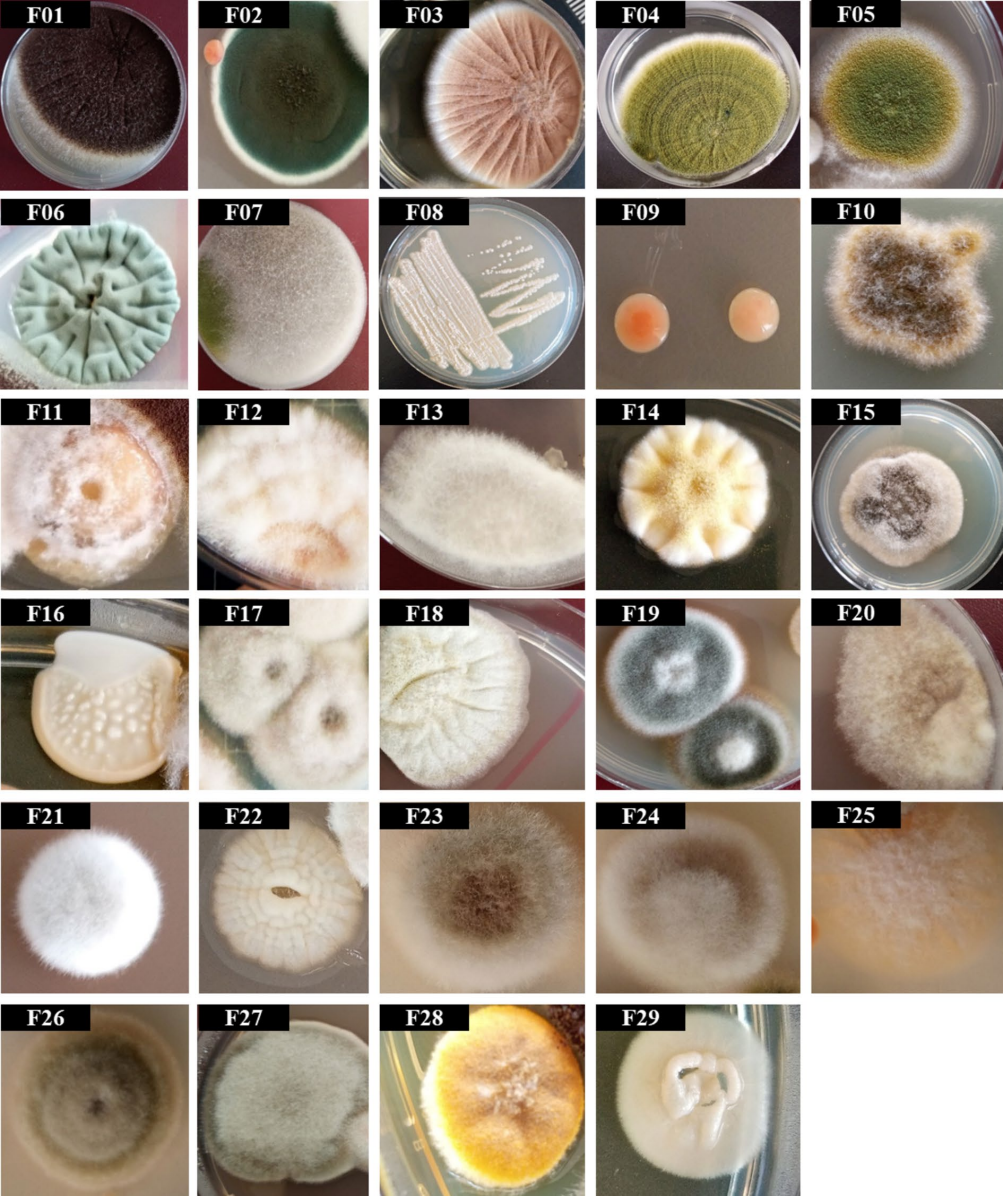
Fungal colonies isolated from the air of the eastern WWTP, Alexandria, Egypt.

According to morphological characteristics, 9 of the 29 isolated fungi colonies (Fig. 6) were identified (only to the genus level)^76–80^, while other isolated fungi colonies require further studies to be identified. Identified colonies were *Aspergillus* spp. (F01, F02, and F03), *Penicillium* spp. (F04, F05, and F06), *Rhizopus* sp. (F07), *Candida* sp. (F08), and *Rhodotorula* sp. (F09). All the identified fungal genera were isolated previously from the air of WWTPs; *Penicillium* spp. and *Aspergillus* spp^51,63,81–84^. , *Rhizopus* sp. and *Rhodotorula* sp^63,85^. , and *Candida* sp^83,85^. Unfortunately, several species of these genera were classified as pathogenic fungi^86^.

## Conclusion

To decrease the gap in the scientific understanding of the overall physical characteristics and measurement of bioaerosols, especially in developing countries, total bacteria, total coliform, and total fungi in the air of a WWTP were monitored by sedimentation method and identified by using 16 S rRNA gene sequencing (for total bacterial isolates), and morphological characteristics (for total coliform and total fungi). The study revealed the following conclusions:

1. High counts were recorded for total bacteria and total fungi in most samples for all sites. The primary clarifiers and grit removal phase recorded the highest mean of total coliform count while other sites recorded limited contamination with coliform. Several bioaerosols, that have been reported before as a cause of human infections, were identified.
2. The absence of an international standard for acceptable limits for pathogenic bioaerosols as all suggested limits related to total bacteria and total fungi which includes several non-pathogenic species.
3. The 16 S rRNA gene sequencing may not be sufficient to guarantee species and strain identity. Morphological tests, biochemical tests, or sequencing of other genes may be needed in parallel with partial 16 S rRNA sequencing for accurate identification of bacterial isolates.

Looking forward to the future, further studies of monitoring bioaerosols are needed to investigate the diversity of bioaerosols by expanding measurements of bioaerosols to include specific measurements of pathogenic bacteria and fungi which were isolated in this study and previous studies, other bioaerosols such as protozoa, viruses, and non-culturable bioaerosols, and other facilities which are expected to be sources of bioaerosols, and hence practical and appropriate control measures can be applied. Moreover, an international standard for monitoring bioaerosols in WWTPs is needed to make the comparison with previous studies more significant. Also, more comparative studies are needed to confirm the accuracy of the Omeliansky formula or to modify/replace it so that measurements using the sedimentation method will be more accurate.

Several precautions can be used to decrease risk of exposure to bioaerosols emitted from the WWTP such as the inactivation of pathogenic bioaerosols in closed workplaces, using of ventilation systems, automating whenever available, applying WWTP main processes only during daylight hours when the inactivation and dilution of airborne pathogens are highest, and considering a safe distance between the WWTP and the residential areas in future urban planning.

## Data Availability

Sequences was deposited at the National Center for Biotechnology Information (NCBI), and their accession numbers are: PQ164272: https://www.ncbi.nlm.nih.gov/nuccore/PQ164272, PQ164273: https://www.ncbi.nlm.nih.gov/nuccore/PQ164273, PQ164282: https://www.ncbi.nlm.nih.gov/nuccore/PQ164282, PQ164283: https://www.ncbi.nlm.nih.gov/nuccore/PQ164283, PQ164289: https://www.ncbi.nlm.nih.gov/nuccore/PQ164289, PQ164339: https://www.ncbi.nlm.nih.gov/nuccore/PQ164339, PQ164341: https://www.ncbi.nlm.nih.gov/nuccore/PQ164341, PQ164346: https://www.ncbi.nlm.nih.gov/nuccore/PQ164346, PQ164347: https://www.ncbi.nlm.nih.gov/nuccore/PQ164347.
